# Fournier’s Gangrene in Patients with Oncohematological Diseases: A Systematic Review of Published Cases

**DOI:** 10.3390/healthcare9091123

**Published:** 2021-08-30

**Authors:** Massimiliano Creta, Antonello Sica, Luigi Napolitano, Giuseppe Celentano, Roberto La Rocca, Marco Capece, Armando Calogero, Gianluigi Califano, Luigi Vanni, Francesco Mangiapia, Davide Arcaniolo, Lorenzo Spirito, Ferdinando Fusco, Marco De Sio, Ciro Imbimbo, Vincenzo Mirone, Caterina Sagnelli, Nicola Longo

**Affiliations:** 1 Department of Neurosciences, Reproductive Sciences and Odontostomatology, University of Naples Federico II, 80130 Naples, Italy; luiginap89@gmail.com (L.N.); dr.giuseppecelentano@gmail.com (G.C.); roberto.larocca@unina.it (R.L.R.); drmarcocapece@gmail.com (M.C.); gianl.califano2@gmail.com (G.C.); mangiapippo@libero.it (F.M.); lorenzospirito@msn.com (L.S.); ciro.imbimbo@unina.it (C.I.); mirone@unina.it (V.M.); nicola.longo@unina.it (N.L.); 2 Department of Precision Medicine, University of Campania “Luigi Vanvitelli”, 80123 Naples, Italy; antonello.sica@fastwebnet.it; 3 Department of Advanced Biomedical Sciences, University of Naples Federico II, 80130 Naples, Italy; armando.calogero2@unina.it; 4 Department of Public Health, University of Naples Federico II, 80130 Naples, Italy; luigi.vanni@libero.it; 5 Department of Woman, Child and General and Specialized Surgery, Urology Unit, University of Campania Luigi Vanvitelli, 81100 Naples, Italy; davide.arcaniolo@gmail.com (D.A.); ferdinando.fusco@unicampania.it (F.F.); marco.desio@unicampania.it (M.D.S.); 6 Department of Mental Health and Public Medicine, University of Campania “Luigi Vanvitelli”, 80131 Naples, Italy; caterina.sagnelli@unicampania.it

**Keywords:** Fournier’s gangrene, necrotizing fasciitis, oncohematology

## Abstract

Patients suffering from hematological malignancies are at increased risk of Fournier’s gangrene (FG) due to immunosuppression caused by the disease itself or by disease-related treatments. A systematic review of PubMed, ISI Web of Knowledge, and Scopus databases was performed in June 2021. We included full papers that met the following criteria: original research, human studies, and describing clinical presentation, treatment, and outcomes of FG in patients with oncohematological diseases. We identified 35 papers published from 1983 to 2021 involving 44 patients (34 males, 8 females) aged between 4 days and 83 years. The most common malignant hematological disorders were acute myeloid leukemia (*n =* 21) and acute lymphocytic leukemia (*n =* 9). In 10 patients FG represented the first presentation of hematological malignancy. Scrotum (*n*= 27) and perineum (*n =* 11) were the sites most commonly involved. *Pseudomonas aeruginosa* (*n =* 21) and *Escherichia coli* (*n =* 6) were the most commonly isolated microorganisms. Surgery was performed in 39 patients. Vacuum-assisted closure and hyperbaric oxygen therapy were adopted in 4 and in 3 patients, respectively. Recovery was achieved in 30 patients. FG-related mortality was observed in 11 patients. FG should be carefully considered in patients with oncohematological diseases.

## 1. Introduction

Fournier’s gangrene (FG) is an acute, rapidly progressive, and potentially fatal infective necrotizing fasciitis involving the perineal, genital, and perianal regions first described in 1883 by the dermatologist and venereologist Jean Alfred Fournier [1]. It represents a rare condition with an overall incidence of 1.6 cases per 100,000 males and accounts for about 0.02% of hospital admissions [2]. The average age of FG patients is 50.9 years and the ratio of men to women is 10:1 [3]. Commonly, the disease is caused by a polymicrobial infection involving the soft tissues of the perineum, the perianal region, and external genitalia [4]. Predisposing factors include advanced age, recent perirectal or perineal surgery, diabetes mellitus, malignancies, perineal trauma or infection, immunocompromised status, and chronic alcoholism. In recent years an increased incidence of FG has been reported, most likely due to an increase in the mean age of the population, as well as increased number of immunocompromised patients [5]. Subjects suffering from hematological malignancies represent a high prevalent subgroup of immunocompromised patients as the incidence of hematological malignancies has recently been evaluated in Europe to be about 230,000 new cases per year and are at increased risk of FG [6,7,8,9,10]. Evidence exist suggesting that necrotizing fasciitis in hematological patients may represent a different scenario if compared to non-hematological ones and that it may pose more challenges due to the immunocompromised status [10]. However, data about FG, a subtype of necrotizing fasciitis, in this subgroup of patients are scarce. In 2013, D’Arena et al. performed a review of the scientific literature focusing on the topic of FG complicating hematologic malignancies by identifying 35 cases [11]. Since then, other cases of FG in patients with oncohematological diseases have been described. Herein, we performed an updated systematic review of the literature aimed at summarizing clinical presentation, treatment, and outcomes of FG in patients with oncohematological diseases.

## 2. Materials and Methods

This review conforms to the “Preferred Reporting Items for Systematic Reviews and Meta Analyses” (PRISMA) statement [12].

### 2.1. Literature Search

The search was performed in the Medline (US National Library of Medicine, Bethesda, MD, USA), Scopus (Elsevier, Amsterdam, The Netherlands), and Web of Science Core Collection (Thomson Reuters, Toronto, ON, Canada) databases up to June 2021. The following terms were combined to capture relevant publications: “Fournier’s Gangrene” OR “Necrotizing fascitis” AND (“hematology” OR “lymphoma” OR “leukemia” OR *“myelodysplasia”* OR *“monoclonal gammopathy” AND “bone marrow transplantation”).* Reference lists in relevant articles and reviews were also screened for additional studies.

### 2.2. Selection Criteria

Two authors (M.Ca. and G.Ce) reviewed the records separately and individually to select relevant publications, with any discrepancies resolved by a third author (C.I.). To assess the eligibility for the systematic review, PICOS (participants, intervention, comparisons, outcomes, study type) criteria were used. PICOS criteria were set as follows: (P)articipants—Patients with hematological malignancy experiencing FG; (I)ntervention—none; (C)omparator—none; (O)utcome: clinical presentation, treatment strategies, survival; (S)tudy types—prospective and retrospective studies, case series, case reports.

### 2.3. Data Collection

The following data were extracted: first author, year of publication, study type, patients’ age and gender, oncohematological disease, relevant comorbidities, underlying urological conditions, FG location, clinical presentation, time from chemotherapy or stem cell transplantation or corticosteroid therapy to FG presentation, complications, microbiological aetiology, white blood cells (WBC) count, neutrophil count, Fournier Gangrene Severity Index (FGSI), empirical and culture-based antimicrobial treatments, surgical treatments, other treatments, outcomes, time from presentation to outcomes.

The methodological quality of case reports and case series was performed according to Murad et al. [13]

## 3. Results

**Figure 1:**
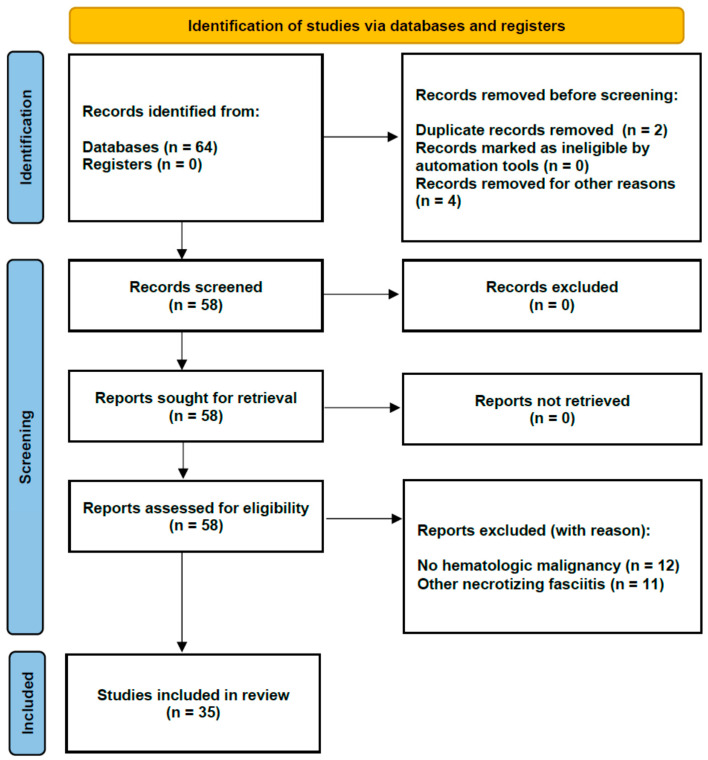
Flow diagram of the systematic review.

The search strategy revealed a total of 64 results. Screening of the titles and abstracts revealed 58 papers eligible for inclusion. Further assessment of eligibility, based on full-text papers, led to the exclusion of nineteen papers. Finally, 35 papers (6 case series and 29 case reports) involving a total of 44 patients were included in the final analysis (Figure 1) [14,15,16,17,18,19,20,21,22,23,24,25,26,27,28,29,30,31,32,33,34,35,36,37,38,39,40,41,42,43,44,45,46,47,48].

Study characteristics and patient’s clinic-demographic profile are reported in Table 1.

Overall, 34 patients were male, and eight were female. In two studies, patients’ sex was not available. Patients’ age ranged from 4 days to 83 years. Twenty-eight patients were aged ≥18 years, 14 patients were aged <18 years, and age was not available in two cases. Acute myeloid leukemia (AML) (*n =* 21) and acute lymphocytic leukemia (ALL) (*n =* 9) were the most frequent oncohematological conditions reported. FG represented the first manifestation of hematological malignancy in 10 patients.

FG was diagnosed in patients under chemotherapy in 18 cases (40.9%). In these patients, FG was observed after a mean of 14.4 days (range: 2–25) from the start of treatment. In four cases (9.1%), patients had received stem cell transplantation. In these patients, FG was observed after a mean of 10 days (range: 8–13) from the transplant. WBC and/or neutrophil count at onset was available in 32 patients. A condition of leukopenia (WBC less than 4000/mm^3^) was reported in 26/32 patients (81.2%).

Scrotum (*n =* 27, 84.3%) was the most frequent involved site followed by perineum (*n =* 11, 34.3%). The penis was involved as a single site in one patient and in combination with the scrotum in eight patients. Fever (*n =* 21, 55.2%), followed by swelling (*n =* 12, 31.5%) and pain (*n =* 15, 39.4%) were the most frequent presenting symptoms.

Microbiological aetiology was available for 34 patients (77.2%). A monomicrobial infection was reported in 22 patients. *Pseudomonas aeruginosa* was the most frequently involved microorganism (*n =* 22/34, 64.7%). It was identified in 18 cases of monomicrobial infection and four cases of polymicrobial infection. Pseudomonas aeruginosa was isolated in 16/26 (61.5%) patients with leukopenia. *Escherichia coli* was the second most commonly involved microorganism (*n =* 6/34, 17.6%)). Table 2 describes details about treatments.

Details about antibiotics prescribed as first-line empirical therapy were provided for 27 patients (61.3%). Combination therapy was used in 23 patients. Aminoglycosides (n= 16) followed by cephalosporines (*n =* 12), glycopeptides (*n =* 10), and lincosamides (*n =* 7) were the drug classes most frequently prescribed in this setting. Details about antibiotics prescribed on the basis of blood culture were provided for 14 patients (31.8%). Combination therapy was used in 12 patients. Aminoglycosides (n= 5), cephalosporines (*n =* 5), carbapenems (*n =* 5), polymyxins (*n =* 4), and glycopeptides (*n =* 4) were the drug classes most frequently prescribed in this setting. Surgical debridement was performed in 38 (86.3%) patients, with more complex surgical procedures being required in 19 (patients. Hyperbaric oxygen therapy (HOT) and vacuum-assisted closure (VAC) were used in three (6.8%) and four (9.1%) patients, respectively. The outcome of FG was available in 41 patients. Gangrene resolution was observed in 30 patients (73.1%) after a mean of 81.6 days (range: 6–1095). The remaining patients (11.26.8%) deceased after a mean of 11.72 days (range: 1–101). The methodological quality of studies included is described in Table 3.

## 4. Discussion

FG is a specific form of necrotizing fasciitis localized on the external genitalia and in the perianal region. It is characterized by gangrene of the skin and subcutaneous tissue with a fulminating course and is associated with a high mortality rate [1]. Typically, FG is more prevalent in adults with peak age between 50 and 79 years and in males with estimates of male-to-female ratio ranging from 10:1 up to 40:1 [3]. Immunosuppression is a recognized risk factor for necrotizing fasciitis. Therefore, patients with hematological malignancies represent a group of patients at increased risk of this infective condition due to immunocompromised status secondary to the disease itself or disease-related treatments [6,7,8,9,10]. The first case of FG associated with a hematological malignancy was reported in 1983 by Patrizi et al. [14]. The diagnosis and therapy of FG may represent a challenge in oncohematological patients.

In line with evidence from the general population, we found a higher prevalence in adults males even in the subset of oncohematological patients. However, despite being especially uncommon in the pediatric age group, FG has been reported in a relevant percentage of pediatric oncohematological patients [9].

Although FG has been reported to occur in several oncohematological diseases, AML followed by ALL represents the most frequent conditions associated with it. Although a pathophysiological relationship between hematological malignancy subtype and FG incidence cannot be identified, the evidence that AML is the most common acute leukemia in adults may be responsible for this observation [8].

The microbiology of FG is often polymicrobial with a predominance of Gram-negative pathogens and other organisms colonising the perineum such as *Escherichia coli*, *Streptococcus* spp., and *Bacteroides* spp. [1,2,3]. Pseudomonas aeruginosa has been reported in about 20% of FG patients [34]. Patients with underlying hematological diseases are known to have multiple hospital encounters and admissions; therefore, they are at a higher risk of exposure to nosocomial, multidrug-resistant organisms [10]. Albasanz-Puig A. et al. performed the first study to compare the characteristics of necrotizing fasciitis between haematological and non-haematological patients [10]. Their results show that monomicrobial necrotizing fasciitis in patients with haematological malignancies is mainly caused by Gram-negative bacteria [10]. In line with this evidence, we found a higher prevalence of Gram-negative bacteria with *Pseudomonas aeruginosa* and *Escherichia coli* being the most frequently isolated pathogens. Interestingly, in patients with oncohematological diseases, monomicrobial *Pseudomonas aeruginosa* infection is more common than in the general population of FG patients. Accordingly, *Pseudomonas aeruginosa* infection is one of the most frequent infections in patients in the agranulocyte state of hematological malignancies [34].

The typical clinical features of FG include sudden pain and swelling in the scrotum, purulent wound discharge, crepitation, fluctuance, prostration, pallor, and a fever greater than 38 °C [5]. Some authors suggest that the inability to mount an appropriate inflammatory response may lead to altered clinical manifestations [10]. However, in line with evidence from the general population of FG patients, we found fever, swelling, and pain to be the most frequent presenting symptoms even in oncohematological patients.

Typically, FG occurs in the neutropenic phase following chemotherapy or stem cell transplantation. However, in some cases, it may represent the first manifestation of a hematologic malignancy. Accordingly, the majority of FG patients identified in the present review had leukopenia.

The key management of FG lies in a high index of suspicion, early diagnosis, appropriate antimicrobial therapy, and early surgery [1].

European Association of Urology Guidelines strongly recommends immediately starting treatment for FG on presentation with empiric parenteral broad-spectrum antibiotics that cover all probable causative organisms and can penetrate inflammatory tissue [4]. A suggested regime would include a third-generation cephalosporin or broad-spectrum penicillin, gentamicin, metronidazole, or clindamycin [4]. Subsequent refinement should be done according to culture and clinical response [4]. Accordingly, results from studies providing details about the antibiotic therapy used as an empirical first-line regimen demonstrate that combination therapy was used in most cases and that aminoglycosides, cephalosporines, glycopeptides, and lincosamides are the drug classes most frequently prescribed in this setting. Unfortunately, details about subsequent antibiotic refinements were available only for a small percentage of patients.

Early and aggressive surgical treatment, often involving multiple debridements with extensive resections, is crucial to improve survival in FG patients [1]. European Association of Urology Guidelines strongly recommends commencing repeated surgical debridement within 24 h of presentation [4].

Some authors have reported lower percentages of surgical treatments in immunocompromised vs. non-immunocompromised patients. Albasanz-Puig A. et al. observed that surgical treatment of necrotizing fasciitis was less common among haematological patients, with only 62.5% undergoing surgery, compared with 100% of non-hematological patients [10]. The fear of high intraoperative mortality due to increased intraoperative bleeding in the context of severe pancytopenia has been hypothesized as one potential explanation for why haematological patients are less likely to undergo surgery [10]. Despite these fears, results from the present review demonstrate that most FG patients with hematological malignancy undergo surgical debridement, with many of them requiring additional surgical procedures. In recent years other treatment options in combination with surgery have been evaluated for the management of FG patients, including HOT and VAC [1].

Several potential benefits of HOT in immunocompromised patients with FG can be hypothesized. Its direct antibacterial activity, the stimulation of intracellular antibiotic transport, the improved phagocytic action of neutrophils, the reduced toxicity of endotoxins, the increased proliferation of fibroblasts, the stimulation of angiogenesis, and the reduction of edema may be of benefit mainly in immunocompromised subjects characterized by an impaired humoral and cell-mediated immunity [49]. Moreover, HOT may restore antibiotic susceptibility by inducing aerobic metabolism. This mechanism has been demonstrated mainly in models involving Pseudomonas aeruginosa, the microorganism most frequently involved in oncohematological patients [49]. Finally, the beneficial role of HBOT in reducing mortality due to FG reported by many authors together with the lack of significant side-effects make this procedure especially useful for particularly fragile patients [49].

Interestingly, the adoption of these treatment strategies has been described in only a small percentage of FG patients with hematological malignancies.

FG-related mortality rates in patients with FG range from 4% to 88% and have been reported to be between 20% and 40% in most cases [1]. Although higher mortality rates have been hypothesized to occur in immunocompromised FG patients, the available evidence in patients with oncohematological diseases demonstrates that FG-related mortality is in line with evidence obtained in unselected FG patients [1].

Results from the present systematic review have relevant clinical implications. Patients suffering from oncohematological diseases should be considered at risk of developing FG mainly during the neutropenic phase following chemotherapy or stem cell transplantation. On the other hand, hematological malignancy should be ruled out in patients presenting with FG, as it has been reported as the first manifestation in some cases. Moreover, despite the well-known aggressivity of FG and the oncohematological comorbidity, a high recovery rate has been reported; thus, emphasizing the need to ensure adequate FG-directed medical and surgical strategies, possibly in a multidisciplinary setting. The high prevalence of gram-negative bacteria, mainly Pseudomonas aeruginosa, should be considered in the context of initial empirical antimicrobial therapy.

However, these data should be considered with caution. The major limitation of the present study derives from the methodological quality of available data. Indeed, data retrieved only derive from case series and case reports that are typically considered the lowest level of evidence in the hierarchy of evidence. However, they are considered to fill an important role as the initial data source for rare and heterogeneous conditions such as FG in oncohematological patients [50]. Although methodological challenging and burdened with a high risk of bias, systematic reviews of case reports and case series can provide a useful addition to evidence-based medicine and can provide the basis for hypothesis generation [50]. Unfortunately, the heterogeneity of published cases and the wide timeframe covered does not allow us to make adequate comparisons in terms of treatment strategies and outcomes. Efforts to publish further evidence about FG in oncohematological patients by adopting standardized reporting systems such as the CAse REport (CARE) checklist are required to improve the evidence level [50].

## 5. Conclusions

Evidence from case reports and case series suggests that FG can occur in patients suffering from oncohematological diseases mainly in the neutropenic phase following chemotherapy or stem cell transplantation. In some cases, it may represent the first manifestation of a hematological malignancy. Gram-negative bacteria, mainly *Pseudomonas aeruginosa*, represent the most frequent aetiological factor. In most cases, recovery is observed, thereby emphasizing the need to ensure adequate FG-directed medical and surgical strategies.

## Figures and Tables

**Table 1:** Study characteristics and patients’ clinic-pathologic characteristics.

Author, Year	Study Type	Age, Years	Sex	Ematological Disease	Relevant Comorbidity	Underlying Surgical Condition, Type	Site of Infection	Clinical Symptoms at Onset	Time from start of Oncohematological Treatment, Days	FG-Related Complications	Aetiology	WBC Count at Onset (mm^3^)	Neutrophil Count at Onset (mm^3^)	FG Severity Index
Patrizi, 1983 [14]	CR	21	M	APML	None	None	Scrotum + penis + thigh	Fever + scrotal ulcer	15 °	None	*P. aeruginosa* ^a^	<500	n/a	n/a
Joo, 1985 [15]	CR	44	M	ALL	n/a	n/a	n/a	n/a	-	n/a	n/a	n/a	n/a	n/a
Berg, 1986 [16]	CS	16	M	BL	None	None	Scrotum + perineum + gluteum	Fever + inguinal pain and tenderness + necrotic scrotal lesions	2 °	Renal failure + Disseminated Candida Tropicalis	*P. aeruginosa* ^a,b^	<500	n/a	n/a
		25	M	AML	None	None	Scrotum + perineum	Tenderness and erythema of the scrotum and perineum	8 °	None	*P. aeruginosa* ^a,b^	<500	n/a	n/a
Radaelli, 1987 [17]	CS	37	M	AML	None	None	Scrotum + glans	Scrotal pain + swelling and necrotic ulcer in the scrotum and glans	17 °	Partial auto-amputation of the genitalia	*P. rettgeri + P. aeruginosa* ^a,b^	n/a	100	n/a
		14	M	ALL	None	None	Scrotum + preputium	Urogenital pain + massive preputial and scrotal edema + necrotic ulcer of the penis	6 °	Septic shock	Negative	n/a	200	n/a
		19	M	NHL	None	None	Scrotum	Fever + scrotal swelling + pain	15 °	None	*P. aeruginosa* ^a,b^	n/a	300	n/a
		20	M	ALL	None	None	Scrotum + preputium	Fever + massive scrotal and preputial edema	n/a	Septic shock	*P. aeruginosa* ^a,b^	n/a	100	n/a
Martinelli, 1998 [18]	CS	41	M	AML	None	None	Scrotum + perineum	Fever + genital erythema + pain + swelling + crepitation	10 ^#^	None	*P. aeruginosa* ^a,b^	500	n/a	>13
		26	F	AML	None	None	Labium majorum + pubis	Redness and swelling of right labium majorum	13 ^#^	Abscess of the rectus abdominis	*P. aeruginosa* ^a,b^	100	n/a	8
		25	F	AML	None	None	Perineum	Fever + pain + edema + erythema + swelling of the perineal area	10^#^	None	*P. aeruginosa* ^a,b^	600	n/a	9
Lèvy, 1998 [19]	CR	44	M	AML	DM2	None	Scrotum	Small, indurated lesion in the right scrotum	16 °	None	*S. faecalis+* *S. coagulase negative* ^b^	>1000	n/a	n/a
Faber, 1998 [20]	CR	50	M	AML **	None	None	Scrotum + perianal region	Fever + diffuse infiltration of the anal region + bluish scrotum	-	Septic shock	*E. coli* ^b^	10,500	0%	n/a
Duncan, 1992 [21]	CR	3	F	ALL	None	None	Labium + abdominal wall and thigh	Vaginal pain + rush on left labium with extension to the buttocks + lethargy and anorexia	16 °		*P. aeruginosa* ^a,b^	150,000	n/a	n/a
Yumura, 2000 [22]	CR	83	M	BL	None	Biopsy inguinal tumor	Scrotum	Fever + reddened scrotal swelling	21 °	None	n/a	n/a	n/a	n/a
Jaing, 2001 [23]	CR	3	F	ALL **	None	None	Inguinal region + right abdomen	Fever + swelling on the right labium + erythema + tenderness	n/a	Septic shock	*P. aeruginosa* ^a,b^	800	n/a	n/a
Castellini, 2001 [24]	CR	54	M	HL	None	None	Scrotum + perineum	Fever + pain and heat in the inguinal, perineal and scrotum + oedema	6 °	None	n/a	900	n/a	n/a
Islamoglu, 2001 [25]	CR	33	M	AML **	None	None	Scrotum + penis	n/a	-	None	*B. fragilis* ^b^	n/a	n/a	n/a
Yoshida, 2002 [26]	CR	16	M	AML	None	None	Scrotum + penis + perineum + thighs + lower abdomen	Fever + penile swelling + miction pain	25 °	MOF	*P. aeruginosa* ^a,b^	100	n/a	n/a
Bakshi, 2003 [27]	CS	6	M	AML	None	None	Prepuce +scrotum	Ulcer and edema over the prepuce	17 °	None	*P. aeruginosa* ^a,b^	n/a	28	n/a
		10	M	ALL	None	None	Scrotum + penis + suprapubic area	Pain and swelling in the prepuce	13 °	Partial auto-amputation of the external genitalia	*P. aeruginosa* ^a,b^	n/a	52	n/a
		9	M	NHL	None	None	Prepuce +glans	Erythema and tenderness of the penis	14 °	None	n/a	n/a	5	n/a
Virgili, 2005 [28]	CR	7mo	M	ALL	None	None	Lower abdomen + pubis + perineum + buttocks	Fever + perianal erythematous and edematous area with anal erosions and ecchymoses	25 °	Fistula	*P. aeruginosa* ^a,b^	600	4%	n/a
Mantadakis, 2006 [29]	CR	21	M	ALL	None	None	Scrotum	Small necrotic area and edema	17 °	Abdominal lymphangitis, septic shock	*P. aeruginosa* ^b^	4100	n/a	n/a
Terrazzas, 2007 [30]	CR	38	M	AML	None	None	Scrotum	Fever + scrotal edema and erythema	-^#^	Sepsis + encepha lopathy	*P. aeruginosa* ^b^ + *E. coli* ^b^	n/a	n/a	n/a
Lohana, 2007 [31]	CR	70	M	TL	DM 2 + MF	None	Scrotum + left groin	n/a	-	None	*S. aureus* + *E. coli* + *Group B Streptococci* + *Mixed anaerobes* ^b^	n/a	n/a	n/a
Naithani, 2008 [32]	CR	17	M	APML	None	None	Scrotum	Fever + painful scrotal vesicular lesions	15¶	None	*Staphylococcus aureus* + *E. coli* ^b^	2200	n/a	n/a
Oiso, 2010 [33]	CR	51	M	AML **	None	None	Scrotum + penis	Fever + painful and edematous erythema on the scrotum and penis	-	None	*Corynebacterium spp*. ^b^	7800	12%	n/a
Kaya, 2011 [34]	CR	71	M	NHL	None	None	Scrotum	Eczema + skin necrosis	13°	None	*P. aeruginosa* ^a^	n/a	n/a	n/a
Durand, 2011 [35]	CR	53	M	AML	Obesity	None	Scrotum + penis	Blackened eschar extending from the base of the penis to the scrotum	-	None	*Rhizopus* *Microspores* ^b^	3010	33%	n/a
Valizadeh, 2011 [36]	CR	36	M	AML	None	None	Scrotum	Fever + ulcers + swelling + edema	14°	None	n/a	2000	n/a	n/a
Melchionda, 2011 [37]	CR	20 days	F	AML	None	None	Perineum	Perineal mucositis	-	Rectum prolaxation	*P. aeruginosa* ^b^	n/a	n/a	n/a
Ruiz-Tovar, 2012 [38]	CS	n/a	n/a	CML	None	None	n/a	n/a	-	None	n/a	n/a	n/a	n/a
		n/a	n/a	MDS	None	None	n/a	n/a	-	None	n/a	n/a	n/a	n/a
Komninos, 2013 [39]	CR	30	M	BL **	Obesity	None	Scrotum + lower abdomen	Fever + pain + erythema and swelling of the left scrotum and lower abdomen	-	Septic shock	*Staphylococci coagulase* (–) + *Klebsiella* + *Proteus sp*. ^a^	212000	n/a	n/a
D’Arena, 2014 [40]	CR	66	M	MDS	None	Anal fistula	Scrotum + perineum	Fever + perineal discomfort + painful anal, penile and scrotal edema	- ^	None	n/a	n/a	n/a	n/a
Rouzrokh, 2014 [41]	CS	6 yr	M	AML	None	Gluteal cellulitis	n/a	n/a	-	None	*Streptococcus*^a^ *P. aeruginosa* ^b^	2500	67%	n/a
		5 mo.	F	ALL	None	Anal fissure	n/a	n/a	-	MOF	*Streptococcus*^a^ *P. aeruginosa* ^b^	1250	70%	n/a
Foo, 2015 [42]	CR	43	F	BL	None	None	Perianal region	Fever + perianal pain + hematoma	-	None	Negative	30	n/a	n/a
Mosayebi, 2016 [43]	CR	4 days	F	AML **	None	None	External genital area	Fever + necrotic lesion in the perineum with swelling of the labium major	-	DIC	*P. aeruginosa* ^a,b^	2160	5%	n/a
Adachi, 2017 [44]	CR	77	M	MDS **	DM2	None	Scrotum + penis	Fever + perineal discomfort + painful penile, and scrotal edema	-	None	n/a	n/a	n/a	n/a
Furtado, 2018 [45]	CR	38	M	APML **	None	None	Perineum	Groin pain + perineal swelling + tenderness and erythematous area	60	None	*B. thetaiotaomicron* + *C. clostridioforme* + *Diphtheroids* + *E. faecalis* + *E. coli* + *S. agalactiae* ^b^	1000	n/a	n/a
Mostaghim, 2019 [46]	CR	38	M	APML **	None	Scrotal folliculitis	Scrotum + perineum	Fever + edematous area draining feculent and serosanguineous fluid	-	None	*E. coli* + *Enterococcus faecalis* + *Bacteroides thetaiotaomicron* + *Streptococcus agalactiae* + *Clostridium clostridioforme* ^b^	1000	19%	n/a
Louro, 2019 [47]	CR	n/a	n/a	MG	None	None	n/a	n/a	-	None	n/a	n/a	n/a	n/a
Yulizar, 2021 [48]	CR	45	M	CML **	None	Priapism	Scrotum + penis	Pain + darkened penis shaft and scrotum	-	None	n/a	n/a	n/a	n/a

ALL: Acute Lymphoblastic Leukemia; APML: Acute Promyelocytic Leukemia; BL: B-Cell lymphoma; CML: Chronic Myeloid Leukemia; CR: Case Report; CS: Case Series; DIC: Disseminated intravascular coagulation; DM2: Diabetes Mellitus type 2; F: Female; FG: Fournier’s Gangrene; HL: Hodgkin Lymphoma; M: Male; MDS: Myelodysplastic Syndromes; MF: Mycosis Fungoides; MG: Monoclonal Gammopathy; MM: Multiple Myeloma; MOF: Multiorgan Failure; NHL: Non-Hodgkin Lymphoma, TL: T-Cell Lymphoma; WBC: White Blood Cells; *: from stem cell transplantation; ^a^: isolated from blood culture; ^b^: isolated from wound culture; **: FG as the first manifestation of oncohematological disease;.°: from chemotherapy; #: from stem cell transplantation; ^: from steroid therapy; ¶: from All-Trans Retinoic Acid alone.

**Table 2:** Medical and surgical treatments and FG outcomes.

Author	Antimicrobial Therapy				Surgical Therapy	Other Therapies	Outcomes	
	Empirical Antimicrobial Regimen	Duration (Days)	Antimicrobial Regimen Based on Cultures	DURATION (Days)			FG Outcome	Time to Outcome (Days)
Patrizi, 1983 [14]	n/a	n/a	n/a	n/a	Debridement	None	Recovered	42
Joo, 1985 [15]	Clindamycin + Tobramycin + Gentamicin	n/a	n/a	n/a	Debridement	None	Died	n/a
Berg, 1986 [16]	Tobramycin + Cefazolin + Penicillin + Clindamycin	n/a	n/a	n/a	Debridement + cortectomy + suprapubic catheter + colostomy	None	Died	6
	Gentamicin + Cephalothin + Clindamycin	n/a	n/a	n/a	Debridement + colostomy	None	Recovered	n/a
Radaelli, 1987 [17]	Cephalothin + Tobramycin + Cotrimoxazole	n/a	Gentamicin + Colistin	n/a	Debridement + urethrostomy	None	Recovered	n/a
	Cephalothin + Tobramycin + Cotrimoxazole	n/a	Colistin + Carbenicillin + Chloramphenicol + Lincomycin	n/a	Debridement + suprapubic cystostomy	None	Died	36 h
	Amikacin + Carbenicillin + Cotrimoxazole	n/a	Colistin	n/a	Debridement	None	Recovered	6
	Ceftazidime + Amikacin	n/a	n/a	n/a	Debridement	HOT	Recovered	n/a
Martinelli, 1998 [18]	Broad-spectrum antibiotics (n.o.s.)	7	Imipenem 1 g/8 h	28	Debridement	None	Recovered	28
	Amikacin 500 mg/12 h	n/a	Amikacin 500 mg/12 h	n/a	Debridement	None	Recovere	n/a
	Ceftazidime + Gentamicin + Teicoplanin	n/a	Imipenem + Amikacin	n/a	Debridement	None	Recovere	27
Lèvy, 1998 [19]	Piperacillin/Tazobactam + Netilmicin + Vancomycin + Amphotericin B + Metronidazole	7	n/a	0	Debridement	None	Recovered	7
Faber, 1998 [20]	Clindamycin + Penicillin G + Ciproxin	n/a	n/a	n/a	Debridement	None	Died	6 h
Duncan, 1992 [21]	Broad-spectrum antibiotics (n.o.s.)	n/a	Broad-spectrum antibiotics (n.o.s.)	n/a	Incision and drainage of the left labium + left labial resection + colostomy + vesicostomy	None	Recovered	730
Yumura, 2000 [22]	n/a	n/a	n/a	n/a	Debridement	None	Recovered	270
Jaing, 2001 [23]	Oxacillin + Gentamycin	1	Ceftazidime + Amikacin	21	Debridement + fasciotomy + pedicled flap	None	Recovered	n/a
Castellini, 2001 [24]	Gentamicin + Metronidazole	n/a	n/a	n/a	Debridement	HOT	Recovered	n/a
Islamoglu, 2001 [25]	n/a	n/a	n/a	n/a	Debridement	None	Died	n/a
Yoshida, 2002 [26]	Broad-spectrum antibiotics (n.o.s.)	2	0	0	None	None	Died	2
Bakshi, 2003 [27]	Ceftazidime+ Amikacin + Vancomycin	n/a	Imipenem/cilastatin + Amikacin + Vancomycin	n/a	Dressing	None	Recovered	n/a
	Ceftazidime+ Amikacin	n/a	Imipenem/cilastatin + Amikacin	n/a	Debridement + skin allograft + suprapubic cystostomy	None	Recovered	n/a
	Imipenem	n/a	n/a	n/a	Debridement + circumcision	None	Recovered	n/a
Virgili, 2005 [28]	Amikacin + Ceftazidime + Teicoplanin + Metronidazole	n/a	n/a	21	Debridement + temporary colostomy	HOT	Recovered	n/a
Mantadakis, 2006 [29]	Colistin + Chloramphenicol + Tetracycline + Levofloxacin + Ceftazidime + Teicoplanin + Voriconazole	4	Meropenem + Piperacillin/tazobactam + Metronidazole + Linezolid + Voriconazole + Colistin	n/a	Debridement + orchiectomy	None	Died	5
Terrazzas, 2007 [30]	Vancomycin + Imipenem	n/a	n/a	n/a	Debridement + flap rotation and free graft	None	Died	101
Lohana, 2007 [31]	Vancomycin + Meropenem	n/a	n/a	n/a	Debridement + application of irradiated graft	VAC	Recovered	18
Naithani, 2008 [32]	n/a	n/a	n/a	n/a	n/a	None	Recovered	42
Oiso, 2010 [33]	Cefpirome 4 gr/die + Clindamycin 2400 mg/die + cilastatin 2 g	9	Cilastatin + Clindamycin	9	None	None	Recovered	14
Kaya, 2011 [34]	n/a	n/a	n/a	n/a	Debridement + creation of artificial anus	None	Recovered	1095
Durand, 2011 [35]	Piperacillin/tazobactam 4.5 g/6 h + Vancomycin 1 g/12 h + Micafungin 1 g/12 h	7	Piperacillin/tazobactam 4.5 g/6 h + Vancomycin 1 g/12 h + Micafungin 1 g/12 h + Liposomal AmB 8 mg/kg IV daily + micafungin (100 mg IV daily) + Posaconazole (400 mg /12 h)	n/a	Debridement + penectomy, scrotectomy, and bilateral orchiectomy	None	Died	14
Valizadeh, 2011 [36]	Broad-spectrum antibiotics (n.o.s.)	n/a	n/a	n/a	Debridement	None	Recovered	n/a
Melchionda, 2011 [37]	Broad-spectrum antibiotics (n.o.s.)	n/a	0	0	Debridement + anorectoplasty	VAC	Recovered	30
Ruiz-Tovar, 2012 [38]	n/a	n/a	n/a	n/a	n/a	None	n/a	n/a
Komninos, 2013 [39]	Meropenem + Clindamycin	n/a	n/a	n/a	Debridement + skin defect covering	None	Recovered	35
D’Arena, 2014 [40]	Broad-spectrum antibiotics (n.o.s.)	n/a	n/a	n/a	Debridement + reconstructive surgery	None	Recovered	n/a
Rouzrokh, 2014 [41]	Broad-spectrum antibiotics (n.o.s.) Broad-spectrum antibiotics (n.o.s.)	n/a	n/a	n/a	Debridement Debridement	None None	Recovered Died	n/a
Foo, 2015 [42]	n/a	n/a	n/a	n/a	Debridement	None	Recovered	n/a
Mosayebi, 2016 [43]	Vancomycin + Meropenem	n/a	n/a	n/a	n/a	None	Died	n/a
Adachi, 2017 [44]	Broad-spectrum antibiotics (n.o.s.)	n/a	n/a	n/a	Debridement + penectomy + scrotectomy	None	n/a	n/a
Furtado, 2018 [45]	Cephalexin	7	Piperacillin/tazobactam + Vancomycin then oral Metronidazole + Levofloxacin	n/a	Debridement	VAC	Recovered	60
Mostaghim, 2019 [46]	Vancomycin + Piperacillin/tazobactam + Clindamycin	n/a	Vancomycin + Cefepime + Metronidazole	14	Debridement	VAC	Recovered	n/a
Louro, 2019 [47]	n/a	n/a	n/a	n/a	Debridement	None	Recovered	46
Yulizar, 2021 [48]	n/a	n/a	n/a	n/a	Debridement + penectomy	None	n/a	n/a

HOT: Hyperbaric Oxygen Therapy; FG: Fournier’s Gangrene; n.o.s.: not otherwise specified; VAC: Vacuum assisted closure.

**Table 3:** Methodological quality of studies included.

Domain	Selection	Ascertainment		Causality				Reporting
Leading Explanatory Question	1	2	3	4	5	6	7	8
Patrizi, 1983 [14]	-	√	√	√	-	-	√	√
Joo, 1985 [15]	√	√	√	√	-	-	√	√
Berg, 1986 [16]	-	√	√	√	-	-	√	√
Radaelli, 1987 [17]	-	√	√	√	-	-	√	√
Martinelli, 1998 [18]	-	√	√	√	-	-	√	√
Lèvy, 1998 [19]	-	√	√	√	-	-	√	√
Faber, 1998 [20]	-	√	√	√	-	-	√	√
Duncan, 1992 [21]	-	√	√	√	-	-	√	√
Yumura, 2000 [22]	-	√	√	√	-	-	√	√
Jaing, 2001 [23]	-	√	√	√	-	-	√	√
Castellini, 2001 [24]	-	√	√	√	-	-	√	√
Islamoglu, 2001 [25]	-	√	√	√	-	-	√	√
Yoshida, 2002 [26]	-	√	√	√	-	-	-	√
Bakshi, 2003 [27]	-	√	√	√	-	-	√	√
Virgili, 2005 [28]	-	√	√	√	-	-	√	√
Mantadakis, 2006 [29]	-	√	√	√	-	-	√	√
Terrazzas, 2007 [30]	-	√	√	√	-	-	√	√
Lohana, 2007 [31]	-	√	√	√	-	-	√	√
Naithani, 2008 [32]	-	√	√	√	-	-	√	√
Oiso, 2010 [33]	-	√	-	√	-	-	√	√
Kaya, 2011 [34]	-	√	√	√	-	-	√	√
Durand, 2011 [35]	-	√	√	√	-	-	√	√
Valizadeh, 2011 [36]	-	√	-	√	-	-	-	√
Melchionda, 2011 [37]	-	√	√	√	-	-	√	√
Ruiz-Tovar, 2012 [38]	√	√	√	√	-	-	√	√
Komni-s, 2013 [39]	-	√	√	√	-	-	√	√
D’Arena, 2014 [40]	-	-	√	√	-	-	-	-
Rouzrokh, 2014 [41]	√	√	√	√	-	-	√	√
Foo, 2015 [42]	√	√	√	√	-	-	√	√
Mosayebi, 2016 [43]	-	√	√	√	-	-	√	-
Adachi, 2017 [44]	-	√	√	√	-	-	√	√
Furtado, 2018 [45]	-	√	√	√	-	-	√	√
Mostaghim, 2019 [46]	-	√	√	√	-	-	√	√
Louro, 2019 [47]	-	√	√	√	-	-	-	-
Yulizar, 2021 [48]	-	√	√	√	-	-	-	√

Leading explanatory questions: **1.** Does the patient(s) represent(s) the whole experience of the investigator (centre), or is the selection method unclear to the extent that other patients with similar presentations may not have been reported? **2.** Was the exposure adequately ascertained? **3.** Was the outcome adequately ascertained? **4.** Were other alternative causes that may explain the observation ruled out? **5.** Was there a challenge/rechallenge phenomenon? **6.** Was there a dose-response effect? **7.** Was follow-up long enough for outcomes to occur? **8.** Is the case(s) described with sufficient details to allow other investigators to replicate the research or to allow practitioners to make inferences related to their own practice? √: Yes.
